# Mixed-dimensional V_2_CT_x_/Ti_3_C_2_T_x_ composite interlayer to boost electrochemical performance of Li-S batteries

**DOI:** 10.3389/fchem.2022.1020538

**Published:** 2022-09-30

**Authors:** Weiqi Zhang, Wenchao Zhang, Jing Yao, Huiqing Lu, Xitian Zhang, LiLi Wu

**Affiliations:** Key Laboratory for Photonic and Electronic Bandgap Materials, Ministry of Education, School of Physics and Electronic Engineering, Harbin Normal University, Harbin, China

**Keywords:** polysulfides, sulfur redox kinetics, V_2_CT_x_/Ti_3_C_2_T_x_, separator, shuttling, lithium–sulfur batteries

## Abstract

**Abstract:** A mixed-dimensional V_2_CT_x_/Ti_3_C_2_T_x_ composite interlayer was successfully prepared to tackle severe polysulfide (LiPS) shuttling and sluggish sulfur redox kinetics for high-performance lithium–sulfur batteries. In the unique nanoarchitecture, two-dimensional Ti_3_C_2_T_x_ nanosheets served as a stable skeleton with superb electronic conductivity, good mechanical strength, and high polysulfide adsorption, whereas one-dimensional V_2_CT_x_ nanorods played a crucial role in chemisorbing LiPSs and catalyzing the conversion of LiPSs due to their high polarity and electrocatalysis. With the synergistic effect of V_2_CT_x_ and Ti_3_C_2_T_x_ composite nanostructures, the cells with the mixed-dimensional V_2_CT_x_/Ti_3_C_2_T_x_ composite interlayer showed an impressive long-term cycling stability and small capacity decay rate of 0.062% per cycle over 600 cycles at 1 C and exhibited an outstanding rate capability of 935.3 mAh·g^−1^ at 2 C.

## Introduction

Lithium–sulfur batteries (LSBs) have been regarded as one of the most promising next-generation high-energy storage devices. This is because of their advantages of possessing high energy density (2,600 Wh·kg^−1^) and being low cost and environmentally friendly (Chung et al., 2018; Ye et al., 2020; Xia et al., 2021). However, the notorious shuttle effect of lithium polysulfides (LiPSs) leads to the loss of active materials, the sluggish LiPS redox kinetics, and the inferior cycling performance. These could limit the specific capacity and cycling lifetime of LSBs (Chen et al., 2019; Tian et al., 2020).

It is important that the severe shuttle effect and sluggish LiPS redox kinetics are overcome to obtain high electrochemical performance of LSBs. Among the effective strategies, the high-performance interlayers between the cathode and separator are proposed. Among the various materials, the carbon-based materials are extensively applied to prepare the interlayer because they have excellent electrical conductivity (Ma et al., 2020; Wei et al., 2020; Yu et al., 2020). However, the weak interaction between nonpolar carbon materials and LiPSs is difficult to block the diffusion of LiPSs to Li metal anode. Therefore, some polar materials, such as metal oxides (Guo et al., 2019; Li et al., 2020), sulfides (Paolella et al., 2018; Yao et al., 2018), nitrides (Yao et al., 2020), and metal–organic frameworks (Wu et al., 2019), are used to prepare interlayers to further chemically anchor LiPSs. Although they can well immobilize the LiPSs and hinder the shuttle effect, their poor electrical conductivity slows down the multistep redox reactions of sulfur (S) species. Recent research studies showed that a catalyst could make the conversion of LiPSs become fast and shorten the residence time of LiPSs in an electrolyte (Song et al., 2019; Zhang et al., 2020). Therefore, if the incorporated interlayers have an excellent electrical conductivity and could anchor the LiPSs as well as catalyze the LiPS conversion, the slow LiPS redox kinetics and the notorious shuttle effect of the LiPSs could be effectively resolved. Thus, the high-performance interlayer was constructed with a rational design. As a representative, post-graphene two-dimensional (2D) black phosphorus (BP) (Hu et al., 2020) and MXenes (Naguib et al., 2011; Gao et al., 2020; Gao et al., 2021a; Gao et al., 2021b; Zhang et al., 2021; Gao et al., 2022a; Gao et al., 2022b; Cao et al., 2022) have attracted tremendous attention since 2011, due to the layered structure and excellent physicochemical properties. Ti_3_C_2_T_x_, a typical MXene material, has been widely used as interlayer materials in LSBs (Ghidiu et al., 2014; Liang et al., 2015; Song et al., 2020). Compared with the carbon-/metal-based materials, Ti_3_C_2_T_x_ possesses good conductivity for electron transport and high mechanical properties for structure stability. Nevertheless, 2D Ti_3_C_2_T_x_ nanosheets tend to aggregate, which will lead to a decrease in active sites for trapping and catalyzing LiPSs, and slow down the ion transport as well (Qiu et al., 2020). One-dimensional (1D) V_2_CT_x_ nanostructures has been recently explored, which possesses the advantages of strong adsorption to LiPSs and catalytic properties (Wu et al., 2020; Zhang et al., 2022). Therefore, mixed-dimensional V_2_CT_x_/Ti_3_C_2_T_x_ nanostructures together as interlayers will achieve high electrochemical performance for LSBs.

Herein, we construct a mixed-dimensional V_2_CT_x_/Ti_3_C_2_T_x_ composite interlayer on separators for LSBs. It can anchor LiPSs by strong chemisorption and accelerate their redox kinetics under the existence of V_2_CT_x_ catalyst. Based on the unique structure, the LSBs achieve an excellent rate capacity of 935.3 mAh·g^−1^ at 2 C. Also, a low-capacity rate decay of 0.062% is obtained after 600 cycles at 1 C. These results illustrate that the incorporation of the multifunctional interlayer is a direct and effective method to achieve high electrochemical performance for LSBs.

## Materials and methods

### Preparation of mixed-dimensional V_2_CT_x_/Ti_3_C_2_T_x_ interlayers

One-dimensional V_2_CT_x_ nanorods and two-dimensional Ti_3_C_2_T_x_ nanosheets were synthesized based on the previous reports (Qiu et al., 2020; Zhang et al., 2022). The suspensions of 1D V_2_CT_x_ nanorods and 2D Ti_3_C_2_T_x_ nanosheets were mixed at a mass ratio of 2:8. Then, saturated LiCl solution was added to the resultant mixed solution and stirred, which led to the mixed V_2_CT_x_/Ti_3_C_2_T_x_ nanostructures settled at the bottom of the container by negatively charge-induced self-assembly (Ghidiu et al., 2014; Naguib et al., 2015). Then, the sediment was washed to remove needless salt. Finally, the V_2_CT_x_/Ti_3_C_2_T_x_ powder was obtained by vacuum-drying. Subsequently, V_2_CT_x_/Ti_3_C_2_T_x_ and PVDF were dissolved in NMP solution at a mass ratio of 9:1, and then the mixed solution was filtered on a Celgard separator (PP) under vacuum. The obtained thin film is called the mixed-dimensional V_2_CT_x_/Ti_3_C_2_T_x_ composite interlayer (the corresponding separator is labelled as V_2_CT_x_/Ti_3_C_2_T_x_-PP). The V_2_CT_x_ and Ti_3_C_2_T_x_ interlayers were prepared using the same procedure. The corresponding separators are labelled as V_2_CT_x_-PP and Ti_3_C_2_T_x_-PP, respectively.

### Li_2_S_6_ adsorption experiment

Li_2_S_6_ solution of 0.01 M was prepared by the chemical reaction of Li_2_S and S at a molar ratio of 1:5 in a 1, 3-dioxolane (DOL) and 1, 2-dimethoxyethane (DME) mixture (1:1 v/v) at 60°C. A volume of 12 mg of Ti_3_C_2_T_x_ and V_2_CT_x_/Ti_3_C_2_T_x_ was added into 1 ml of Li_2_S_6_ solution, and then, the solutions were rested for adsorption.

### Assembly of symmetric cells

Ti_3_C_2_T_x_ and V_2_CT_x_/Ti_3_C_2_T_x_ (w/w 2:8) were dissolved in alcohol solution. The resulting solutions were dripped onto the carbon paper (CP) with a diameter of 15 mm (labelled as Ti_3_C_2_T_x_-CP and V_2_CT_x_/Ti_3_C_2_T_x_-CP). Both mass loadings were ∼0.50 mg·cm^−2^. The 2025-type coin cells with two Ti_3_C_2_T_x_-CP or V_2_CT_x_/Ti_3_C_2_T_x_-CP electrodes as the anode and cathode were assembled, using PP and 0.1 M of Li_2_S_6_ as the electrolyte.

### Nucleation of Li_2_S on Ti_3_C_2_T_x_ and V_2_CT_x_/Ti_3_C_2_T_x_

First, 0.25 M of Li_2_S_8_ solution was obtained by dissolving S and Li_2_S at a molar ratio of 7:1 in a tetraglyme solvent at 60°C overnight. Following this, Ti_3_C_2_T_x_ and V_2_CT_x_/Ti_3_C_2_T_x_ were dissolved in alcohol solution to obtain uniform suspensions by ultrasound, which were dripped on CPs (10 mm diameter) and dried at 60°C to obtain electrodes. The coin cell was composed of Ti_3_C_2_T_x_-CP or V_2_CT_x_/Ti_3_C_2_T_x_-CP cathode, lithium anode, and PP. The Li_2_S_8_ electrolyte of 0.25 M was used on the cathode side, and the standard electrolyte was used on the anode side. Then, the cells were galvanostatically discharged to 2.06 V at 0.112 mA and then kept at 2.05 V. Also, Li_2_S nucleated and grew until the current of 10^−2^ A was reached.

## Material characterizations and electrochemical analyses

The morphology of mixed-dimensional V_2_CT_x_/Ti_3_C_2_T_x_ composite interlayers were observed using scanning electron microscopy (SEM) (SU70, Japan). The X-ray diffraction (XRD) measurement was recorded using a Rigaku D/max2600 X-ray diffractometer. The cathode is a mixture of Ketjen black/sulfur, super-P, and PVDF at a mass ratio of 8:1:1. The diameter of the cathode was about 13 mm, and the loading of S was ∼1 mg·cm^−2^. The coin cells were assembled with KB/S cathode, Li anode, and PP with Ti_3_C_2_T_x_-PP, V_2_CT_x_/Ti_3_C_2_T_x_-PP, or V_2_CT_x_-PP, respectively. The electrolyte consisted of 1.0 M lithium bis-trifluoromethane sulfonimide (LiTFSI), 2% LiNO_3_ additives, and a mixture of DOL/DME (volume ratio = 1:1). The galvanostatic charge–discharge (GCD) profile of the assembled cells was tested at 0.5 C in the voltage range of 1.7–2.6 V using the LAND cell testing system. The electrochemical impedance spectra (EIS) and cyclic voltammograms (CV) were carried out using an electrochemical workstation (VMP3). X-ray photoelectron spectroscopy (XPS) was conducted by applying ESCALAB 250XI.

## Results and discussion

**FIGURE 1:**
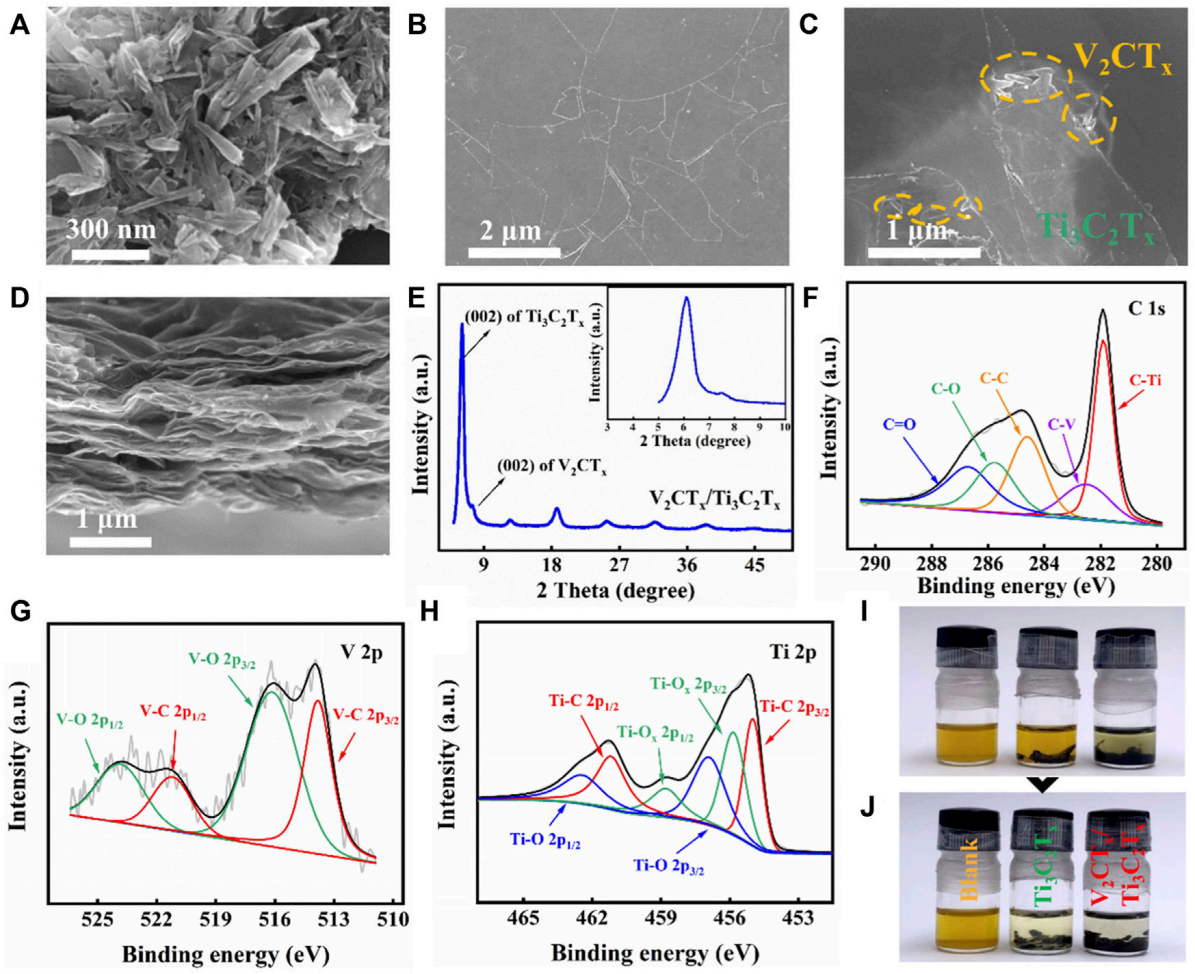
SEM images of **(A)** V_2_CT_x_ nanorods, **(B)** Ti_3_C_2_T_x_ nanosheets, and **(C)** V_2_CT_x_/Ti_3_C_2_T_x_ composite interlayer. **(D)** Cross-sectional SEM image and **(E)** XRD pattern of the V_2_CT_x_/Ti_3_C_2_T_x_ composite interlayer. High-resolution XPS spectra of **(F)** C1s, **(G)** V 2p, and **(H)** Ti 2p of the V_2_CT_x_/Ti_3_C_2_T_x_ composite interlayer. Visualized experiment of Li_2_S_6_: **(I)** initial states and **(J)** final states after 5 h.

SEM images of 1D V_2_CT_x_ nanorods and 2D Ti_3_C_2_T_x_ nanosheets are shown in Figures 1A,B. The length of V_2_CT_x_ nanorods is about 230 nm, while the diameter is tens of nanometers. Also, the size of irregular 2D Ti_3_C_2_T_x_ nanosheets is a few micrometers. Furthermore, the top-view SEM image of the mixed-dimensional V_2_CT_x_/Ti_3_C_2_T_x_ composite interlayer is presented in Figure 1C. The trace amount of V_2_CT_x_ nanorods can be seen on the top surface of the composite interlayer. On the other hand, the cross-sectional SEM image of the V_2_CT_x_/Ti_3_C_2_T_x_ composite interlayer shows that V_2_CT_x_ and Ti_3_C_2_T_x_ are superimposed on each other, forming a well-arranged layered stacking structure (Figure 1D). XRD of the V_2_CT_x_/Ti_3_C_2_T_x_ composite interlayer was carried out to characterize the crystalline structure, as shown in Figure 1E. The two diffraction peaks at 6.1 and 7.45^o^ correspond to the (002) crystal plane of 2D Ti_3_C_2_T_x_ nanosheets and 1D V_2_CT_x_ nanorods, respectively (Li et al., 2017; Zhang et al., 2022). In contrast, the (002) diffraction peak intensity of V_2_CT_x_ is weaker, due to the low content of V_2_CT_x_. The XRD pattern proves that V_2_CT_x_ and Ti_3_C_2_T_x_ were successfully synthesized. The composition of the V_2_CT_x_/Ti_3_C_2_T_x_ composite interlayer was further confirmed by XPS analysis (Li et al., 2017; Zhang et al., 2022). A high-resolution XPS spectrum of C 1s in the V_2_CT_x_/Ti_3_C_2_T_x_ composite interlayer is shown in Figure 1F. C-V and C-Ti bonds were observed. Meanwhile, the corresponding V-C and Ti-C bonds can also be found in the high-resolution XPS spectra of V 2p and Ti 2p in V_2_CT_x_ and Ti_3_C_2_T_x_, respectively, further proving that the composite interlayer is composed of V_2_CT_x_ and Ti_3_C_2_T_x_ (Figures 1G,H). The LiPS adsorption behavior of V_2_CT_x_/Ti_3_C_2_T_x_ composite materials was surveyed by visualized adsorption tests, as shown in Figures 1I,J. Ti_3_C_2_T_x_ was used as the control sample. Figure 1I shows the initial states of different samples placed in Li_2_S_6_ solutions. In Figure 1J, the solvent colors with both Ti_3_C_2_T_x_ and V_2_CT_x_/Ti_3_C_2_T_x_ composite materials undergo a significant change after resting for 5 h. The solution including Ti_3_C_2_T_x_ materials changes only lighter in color. However, the solution including V_2_CT_x_/Ti_3_C_2_T_x_ composite materials become almost colorless. These findings demonstrate the strong chemical adsorption of V_2_CT_x_/Ti_3_C_2_T_x_ composite materials to LiPSs compared with Ti_3_C_2_T_x_.

**FIGURE 2:**
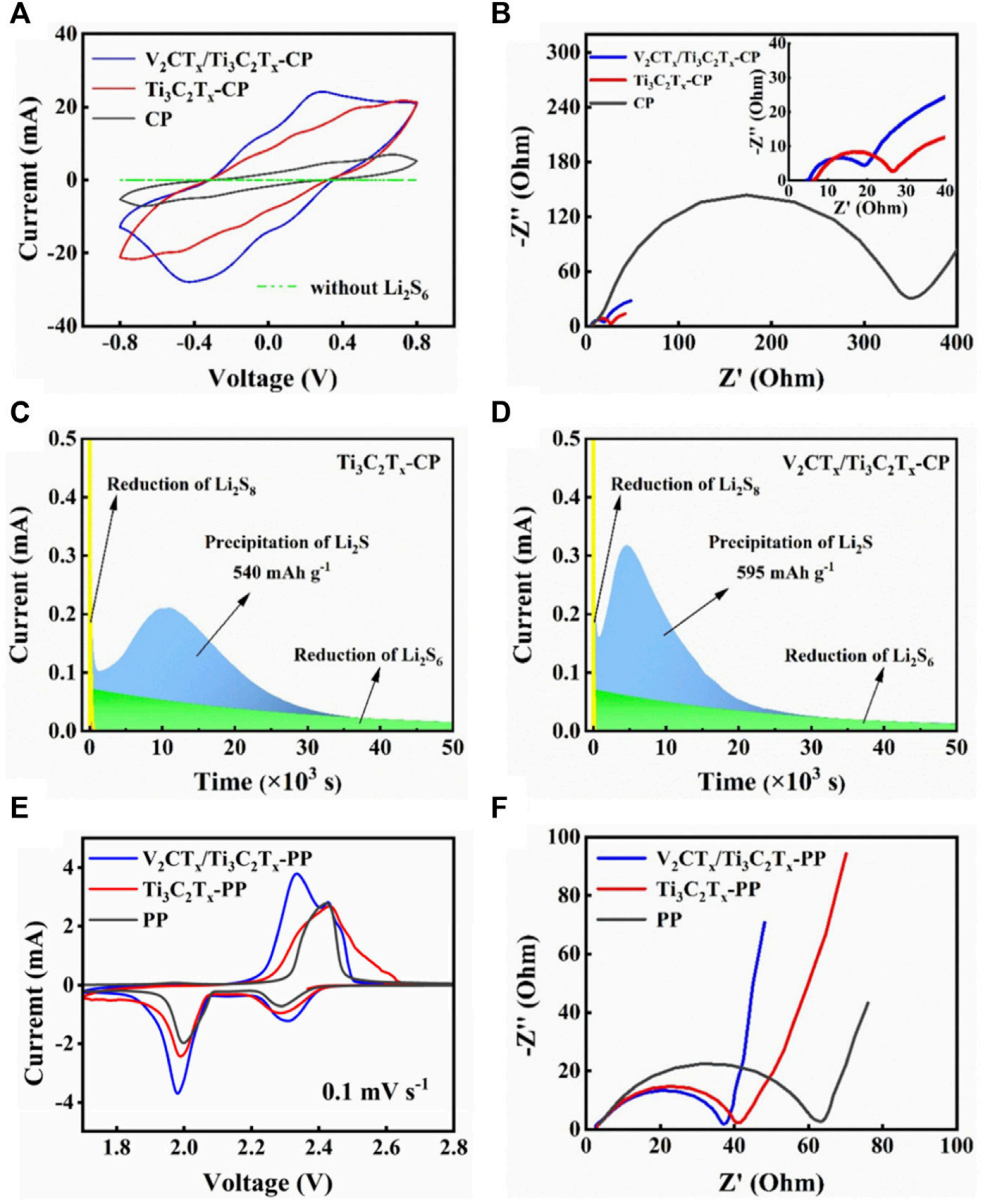
**(A)** CV curves of different symmetric cells under 50 mV·s^−1^ and **(B)** EIS spectra of V_2_CT_x_/Ti_3_C_2_T_x_-CP, Ti_3_C_2_T_x_-CP, and CP symmetric cells using Li_2_S_6_. Current vs. time curves for **(C)** Ti_3_C_2_T_x_-CP and **(D)** V_2_CT_x_/Ti_3_C_2_T_x_-CP electrodes. **(E)** CV curves of PP, Ti_3_C_2_T_x_-PP, and V_2_CT_x_/Ti_3_C_2_T_x_-PP cells at 0.1 mV·s^−1^. **(F)** EIS spectra of PP, Ti_3_C_2_T_x_-PP, and V_2_CT_x_/Ti_3_C_2_T_x_-PP cells.

The mixed-dimensional interlayer with more active sites and stronger catalytic capacity can facilitate the solid–liquid–solid transformations of the S species. At the first stage of LiPS conversions in LSBs, S undergoes solid–liquid phase transformation to high-order LiPSs and then liquid–liquid conversion to low-order LiPSs. Rapid liquid–liquid phase conversions will reduce LiPS accumulation in the electrolyte, which is conductive to ion transport. To investigate the effect of interlayers on the conversions of LiPSs in a liquid–liquid phase, the CV curves of V_2_CT_x_/Ti_3_C_2_T_x_-CP and Ti_3_C_2_T_x_-CP symmetric cells were measured, as shown in Figure 2A. The current response of the CP symmetric cell without Li_2_S_6_ is almost in line. It can be seen from Figure 2A that the current response of V_2_CT_x_/Ti_3_C_2_T_x_-CP symmetric cells with Li_2_S_6_ is the largest among the Ti_3_C_2_T_x_-CP and CP cells, indicating that V_2_CT_x_/Ti_3_C_2_T_x_ composite materials accelerate the liquid–liquid conversion of LiPSs, make the liquid phase LiPS conversion become more thorough, and boost the LiPS redox kinetics. At the same time, the accumulation of LiPSs in the electrolyte will also be greatly reduced. This is due to the rapid conversion of liquid LiPSs. It is beneficial to suppress the shuttle effect. Figure 2B shows the EIS curves of symmetric cells. The smallest semicircle diameter of the V_2_CT_x_/Ti_3_C_2_T_x_-CP symmetric cell implies the smallest charge transfer resistance (R_ct_), thus confirming the rapid electron and ion transportation at the interface between V_2_CT_x_/Ti_3_C_2_T_x_ composite materials and S species. Kinetics of Li_2_S precipitation in LSBs is another significant factor for high-performance LSBs. This is due to the fact that 75% of the capacity originates from the Li_2_S deposition during discharge. Therefore, Li_2_S nucleation tests were carried out. The constant-voltage discharge curves at 2.05 V are shown in Figures 2C,D. Obviously, the V_2_CT_x_/Ti_3_C_2_T_x_-CP electrode demonstrates the earlier Li_2_S deposition compared with Ti_3_C_2_T_x_-CP electrodes, indicating that the V_2_CT_x_/Ti_3_C_2_T_x_-CP electrode has the greater catalytic ability, and accelerates the conversion of LiPSs to Li_2_S. The Li_2_S precipitation capacity on Ti_3_C_2_T_x_-CP and V_2_CT_x_/Ti_3_C_2_T_x_-CP electrodes were calculated by the integral of current vs. time, corresponding to 540 and 595 mAh·g^−1^, respectively. The precipitation capacity of the V_2_CT_x_/Ti_3_C_2_T_x_-CP electrode becomes higher, implying that the faster LiPS conversion is achieved. The PP, Ti_3_C_2_T_x_-PP, and V_2_CT_x_/Ti_3_C_2_T_x_-PP cells were assembled to assess the effect of PP, Ti_3_C_2_T_x_-PP, and V_2_CT_x_/Ti_3_C_2_T_x_-PP on the electrochemical performance of LSBs. Figure 2E shows the CV profiles of PP, Ti_3_C_2_T_x_-PP, and V_2_CT_x_/Ti_3_C_2_T_x_-PP cells at a scan rate of 0.1 mV·s^−1^. Two reduction peaks for three cells can be seen and be attributed to the two reduction processes of S_8_ to LiPSs (Li_2_S_x_, 4 ≤ x ≤ 8) and Li_2_S_4_ to Li_2_S_2_/Li_2_S, respectively. Two oxidation peaks correspond to the oxidation process of Li_2_S to LiPSs, which are then oxidized to S_8_. The CV curve of the V_2_CT_x_/Ti_3_C_2_T_x_-PP cell shows the most intense peak, the lowest electrochemical polarization, and the highest current density, illustrating that the V_2_CT_x_/Ti_3_C_2_T_x_ composite interlayer makes LiPS redox reactions become the fastest among PP and Ti_3_C_2_T_x_-PP cells. In addition, Figure 2F also reveals the excellent electrochemical kinetics of the V_2_CT_x_/Ti_3_C_2_T_x_-PP cell from the EIS curve. The results show that the V_2_CT_x_/Ti_3_C_2_T_x_-PP cell has the lowest R_ct_ and excellent charge transfer ability at the electrolyte/electrode interface. This is due to its high ionic and electronic conductivity.

**FIGURE 3:**
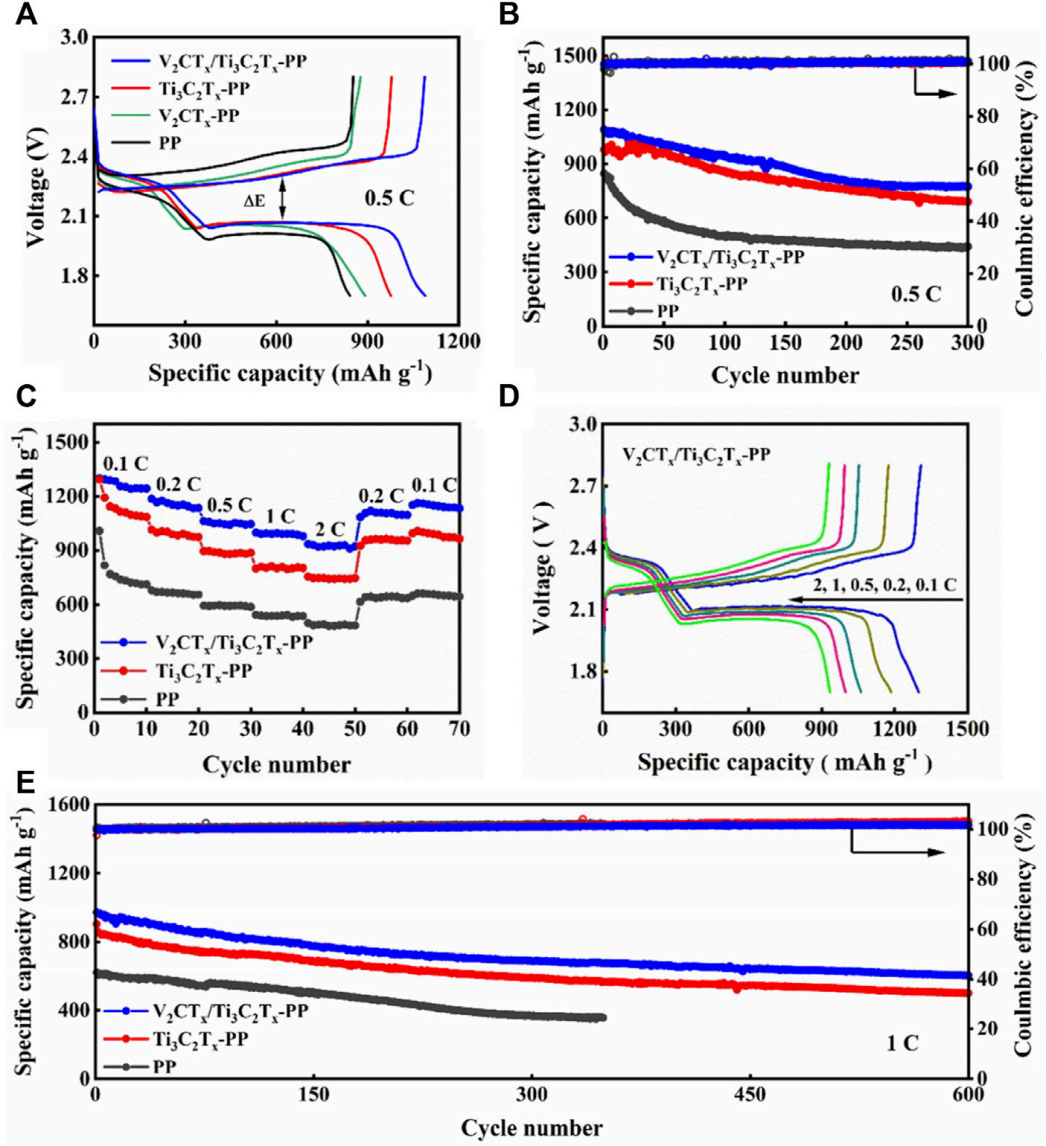
**(A)** GCD curves of V_2_CT_x_/Ti_3_C_2_T_x_-PP, Ti_3_C_2_T_x_-PP, V_2_CT_x_-PP, and PP cells at 0.5 C. **(B)** Cycling performance of V_2_CT_x_/Ti_3_C_2_T_x_-PP, Ti_3_C_2_T_x_-PP, and PP cells at 0.5 C. **(C)** Rate performance of V_2_CT_x_/Ti_3_C_2_T_x_-PP, Ti_3_C_2_T_x_-PP, and PP cells. **(D)** GCD curves of the V_2_CT_x_/Ti_3_C_2_T_x_-PP cell at current densities of 0.1, 0.2, 0.5, 1, and 2 C. **(E)** Cycling performance of V_2_CT_x_/Ti_3_C_2_T_x_-PP, Ti_3_C_2_T_x_-PP, and PP cells at 1 C.

The GCD curves of different cells were measured at 0.5 C, as shown in Figure 3A. The discharge capacities of V_2_CT_x_/Ti_3_C_2_T_x_-PP, Ti_3_C_2_T_x_-PP, V_2_CT_x_-PP, and PP cells are 1,090, 2, 977.3, 892.4, and 842.8 mAh·g^−1^, respectively. In contrast, the V_2_CT_x_/Ti_3_C_2_T_x_-PP cell has the highest specific discharge capacity due to the existence of trace amounts of V_2_CT_x_. In addition, the polarization overpotential of the V_2_CT_x_/Ti_3_C_2_T_x_-PP cell is only 211 mV, which is significantly smaller than that of other cells and consistent with the results of CV tests. Then, the cycling performance tests of the PP, Ti_3_C_2_T_x_-PP, and V_2_CT_x_/Ti_3_C_2_T_x_-PP cells were also performed, as shown in Figure 3B. The V_2_CT_x_/Ti_3_C_2_T_x_-PP cell can still remain at a high discharge capacity of 775.2 mAh·g^−1^ and high capacity retention rate of 71% after 300 cycles. However, the capacity retention rate of Ti_3_C_2_T_x_-PP and PP cells are only 69 and 52% after 300 cycles, respectively. Under the rate performance test shown in Figure 3C, the V_2_CT_x_/Ti_3_C_2_T_x_ cell shows excellent rate performance as the current density changes due to the good ionic and electronic conductivity of the V_2_CT_x_/Ti_3_C_2_T_x_ composite interlayer. In Figure 3C, the discharge capacity of the V_2_CT_x_/Ti_3_C_2_T_x_-PP cell is 1,299.2, 1,185.9, 1,061.8, 998.3, and 935.3 mAh·g^−1^ at 0.1, 0.2, 0.5, 1, and 2 C, respectively. Subsequently, the discharge capacity can be restored to the high reversible capacity of 1,151.8 mAh·g^−1^ by reducing to 0.1 C. However, the discharge capacity of Ti_3_C_2_T_x_-PP and PP cells is 753.9 and 469.2 mAh·g^−1^ at 2 C, respectively, indicating that the capacity decays faster with the increase in current density. Their reversible capacities correspond to 993 and 647.1 mAh·g^−1^ at 0.1 C, respectively. Figure 3D shows the GCD profiles of the V_2_CT_x_/Ti_3_C_2_T_x_-PP cell. The polarization of the V_2_CT_x_/Ti_3_C_2_T_x_-PP cell is only 123.4 mV at 0.1 C. The excellent rate performance and low polarization of the V_2_CT_x_/Ti_3_C_2_T_x_-PP cell can be attributed to the important role of the mixed-dimensional V_2_CT_x_/Ti_3_C_2_T_x_ composite interlayer in electrical conductivity and chemical anchoring. The cycle stability at a high current density is an important factor to evaluate the performance of LSBs. Therefore, the long cycling stability of different batteries were tested at 1 C. In Figure 3E, the V_2_CT_x_/Ti_3_C_2_T_x_-PP cell has an excellent long-term cycling stability with a high initial capacity of 969.9 mAh·g^−1^ and low capacity decay rate of 0.062% after 600 cycles. In contrast, the Ti_3_C_2_T_x_-PP cell has a capacity decay rate of 0.074%. The PP cell is out of service after less than 400 cycles. The V_2_CT_x_/Ti_3_C_2_T_x_ cell demonstrates excellent electrochemical performance and cycle stability. It is also worth noting that the electrochemical performance of the mixed-dimensional V_2_CT_x_/Ti_3_C_2_T_x_ composite interlayer is highly competitive compared with that of the other materials reported (Table 1). In a word, the adsorbed LiPSs on the composite interlayer can quickly obtain electrons and ions at the adsorption sites to continue the redox reactions based on the high conductivity and abundant active sites of V_2_CT_x_/Ti_3_C_2_T_x_. Meanwhile, the catalytic effect of V_2_CT_x_ accelerates the LiPS redox kinetics.

**TABLE 1:** Comparison of the electrochemical performance between this work and other previously reported works.

Nanostructure	S loading (mg cm^2^)	Discharge rate (C)	Final capacity (mAh g^−1^)	Cycle number	Capacity decay per cycle (%)	Reference
V_2_CT_x_/Ti_3_C_2_T_x_-PP	1.0	0.5	775.2	300	0.096	This work
S/Ti_3_C_2_T_x_-N	—	0.5	1,104.3	100	0.138	Qi and Zhang, (2022)
Bi-PP	2.5	0.5	650	200	—	Huang et al. (2020a)
V_2_CT_x_/CNT-PP	1.0	0.5	1008.3	100	0.204	Zhang et al. (2022)
KB/V_2_CT_X_-PP	1.0	0.2	942	150	0.158	Han et al. (2022)

## Conclusion

In conclusion, we construct a mixed-dimensional V_2_CT_x_/Ti_3_C_2_T_x_ composite interlayer to suppress LiPS shuttling and accelerate LiPS redox kinetics. Profiting from the advantages of strong chemisorption of the composite interlayer to LiPSs and catalysis of V_2_CT_x_, the V_2_CT_x_/Ti_3_C_2_T_x_ cell achieves an excellent rate capacity of 935.3 mAh·g^−1^ at 2 C and low capacity rate decay of 0.062% after 600 cycles at 1 C. Meanwhile, designing a mixed-dimensional composite interlayer can provide a route to develop high-performance LSBs.

## Data Availability

The original contributions presented in the study are included in the article/Supplementary Material. Further inquiries can be directed to the corresponding author/s.
